# Glomus Tumors of Uncertain Malignant Potential Arising in the Endobronchus and Lung Parenchyma

**DOI:** 10.7759/cureus.114555

**Published:** 2026-08-14

**Authors:** Hao Ma, Fengzhang Zhu, Shizhou Tang, Jiaxin Yin, Xianglin Zhang

**Affiliations:** 1 Radiology, The First Affiliated Hospital of Jinzhou Medical University, Jinzhou, CHN; 2 Chest Tumors, The First Affiliated Hospital of Jinzhou Medical University, JInzhou, CHN; 3 Lung Cancer, The First Affiliated Hospital of Jinzhou Medical University, Jinzhou, CHN

**Keywords:** bronchoscopy, endobronchial lesion, glomus tumor of uncertain malignant potential, hemoptysis, pulmonary glomus tumor, pulmonary nodule, sleeve lobectomy

## Abstract

Pulmonary glomus tumors are rare, and synchronous endobronchial and pulmonary parenchymal lesions are particularly uncommon. A 72-year-old woman was admitted with a 20-day history of cough and blood-streaked sputum. Contrast-enhanced chest computed tomography demonstrated a 25 × 20 mm solid nodule in the apicoposterior segment of the left upper lobe and a 13 × 8 mm endobronchial nodule in the left upper-lobe bronchus. Both lesions had smooth margins, prominent vascularity, and persistent enhancement. Bronchoscopy showed complete obstruction of the left upper-lobe bronchus by a smooth intraluminal mass. Electrosnare resection with electrocoagulation was performed, and pathology indicated a glomus tumor of uncertain malignant potential. Because a small endobronchial remnant was suspected, the parenchymal lesion showed the same histology, and local treatment could not ensure complete resection, a multidisciplinary team recommended video-assisted thoracoscopic left upper-lobe sleeve resection. The resection specimen contained a 2.5 × 2.5 × 2.3 cm mass adjacent to and protruding into the bronchus. Bronchial margins, visceral pleura, and dissected lymph nodes were free of tumor. Immunohistochemistry showed positivity for cytokeratin, desmin, smooth muscle actin, and vimentin; together with the morphology, these findings supported the diagnosis of glomus tumor. The patient recovered uneventfully and remained clinically stable during the reported follow-up. Hypervascular endobronchial or pulmonary parenchymal nodules with persistent enhancement should prompt consideration of a pulmonary glomus tumor, although definitive diagnosis requires morphologic and immunophenotypic assessment. When pathology indicates uncertain malignant potential with multifocal disease or a risk of residual tumor, treatment should balance oncologic completeness, preservation of pulmonary function, and long-term imaging surveillance.

## Introduction

Glomus tumors are uncommon perivascular soft-tissue neoplasms, accounting for fewer than 2% of soft-tissue tumors. They are generally considered to arise from glomus bodies, specialized thermoregulatory structures. Most occur in the distal fingers or toes; involvement of the lung or tracheobronchial tree is exceptionally rare [1-3]. Pulmonary glomus tumors may arise in the lung parenchyma or airways and have nonspecific clinical and imaging findings. The differential diagnosis commonly includes primary lung carcinoma, tuberculoma, and pulmonary sclerosing pneumocytoma.

We report a pulmonary glomus tumor with synchronous lesions in the left upper-lobe bronchus and pulmonary parenchyma. The key diagnostic and therapeutic issues were recognition of hypervascular imaging features, bronchoscopic acquisition of diagnostic tissue, and selection of definitive surgery in the setting of uncertain malignant potential and a risk of residual local disease. This case is exceptionally rare and holds significant value for both research and clinical education. To definitively rule out the potential for recurrence or malignant transformation, the patient is currently under continuous follow-up at our institution. The patient has provided explicit informed consent and is fully supportive of this ongoing observation.

## Case presentation

Patient information and initial assessment

A 72-year-old woman was admitted with a 20-day history of cough and blood-streaked sputum without an obvious precipitant. She had taken traditional Chinese medicine without symptom relief. Her general condition was good, with no appreciable weight loss. Her temperature was 36.8 °C, pulse 82 beats/min, respiratory rate 24 breaths/min, and blood pressure 155/75 mmHg. The chest was symmetrical; breath sounds were clear bilaterally, without rales or wheezing, and there was no digital clubbing.

Laboratory testing showed a white blood cell count of 6.5 x 10^9^/L, red blood cell count of 4.2 x 10^12^/L, platelet count of 220 x 10^9^/L, prothrombin time of 12.5 seconds, activated partial thromboplastin time of 32.0 seconds, and fibrinogen of 3.0 g/L. No clinically significant abnormality that would preclude subsequent invasive procedures was identified (Table 1).

**Table 1:** Diagnostic and Treatment Timeline.

Phase	Time	Key event
Symptom onset	20 days before admission	Cough and blood-streaked sputum developed without an obvious precipitant; self-administered roxithromycin dispersible tablets and Yunnan Baiyao powder did not relieve symptoms.
Initial evaluation and admission	Day of admission	Contrast-enhanced chest CT identified a solid nodule in the apicoposterior segment of the left upper lobe and a nodule in the left upper-lobe bronchus; the patient was admitted for pulmonary masses.
Bronchoscopic diagnosis and treatment	Hospital day 2	A mass completely obstructed the left upper-lobe bronchial orifice. Electrosnare resection and electrocoagulation were performed; pathology indicated a glomus tumor of uncertain malignant potential.
First post-procedure assessment	After bronchoscopy	Noncontrast chest CT showed a patent left upper-lobe bronchus without a definite endoluminal nodule.
Definitive surgery	Hospital day 18	Video-assisted thoracoscopic left upper-lobe sleeve resection with lymph-node dissection was performed; the parenchymal lesion had the same pathology as the endobronchial lesion.
Early postoperative assessment	Postoperative day 2	CT showed left pleural air and fluid with a drainage catheter, consistent with postoperative change.
Repeat postoperative assessment	Postoperative day 23	CT showed a reduction in pleural air and fluid compared with the earlier examination.
Follow-up	Reported follow-up period	Recovery after both procedures was uneventful; the patient's condition remained stable, with ongoing scheduled surveillance.

Imaging and bronchoscopic examination

**Figure 1:**
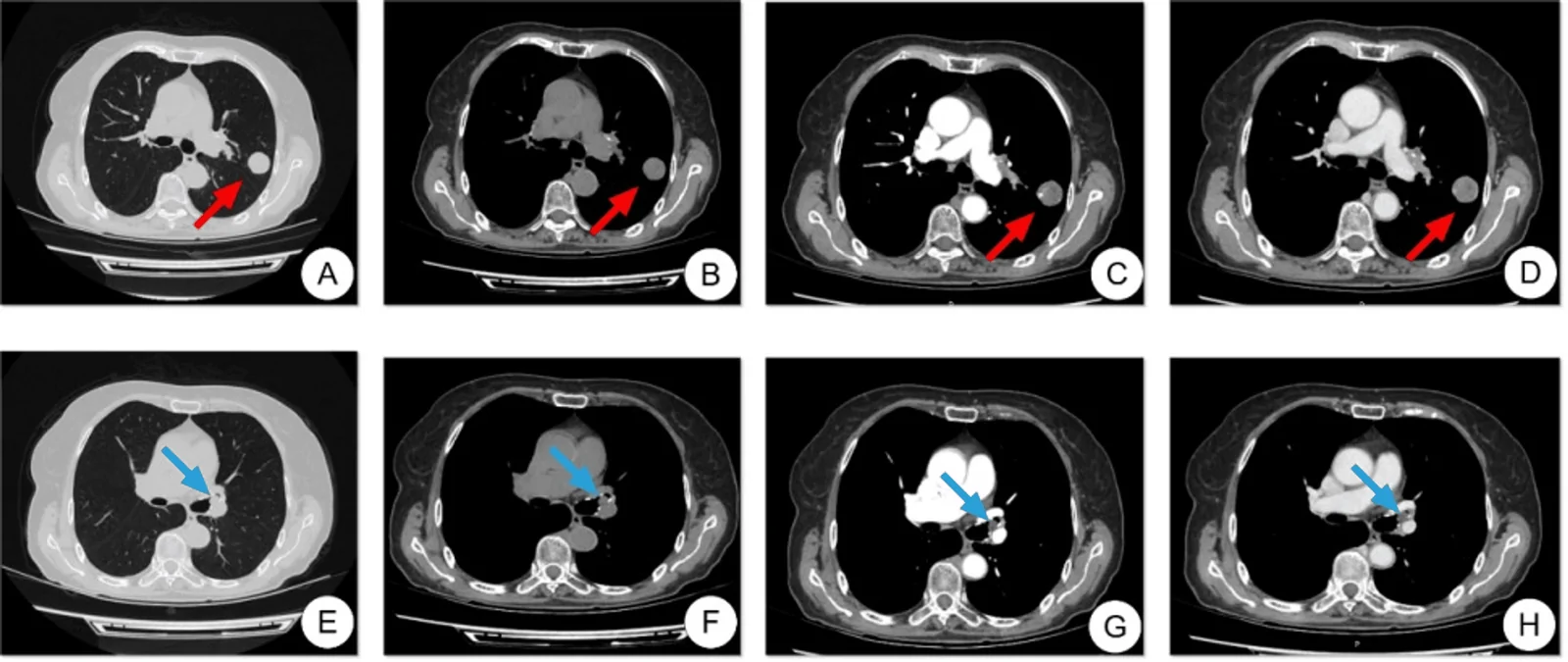
Contrast-Enhanced Chest Computed Tomography. (A-D) Lung-window, mediastinal-window, arterial-phase, and venous-phase images of the nodule in the apicoposterior segment of the left upper lobe. (E-H) Corresponding images of the endobronchial nodule in the left upper-lobe bronchus. The red arrow indicates an intrapulmonary lesion, and the blue arrow indicates an endobronchial lesion.

Contrast-enhanced chest computed tomography obtained before admission revealed two lesions. The first was a 25 x 20 mm solid nodule with smooth margins in the apicoposterior segment of the left upper lobe; multiple tortuous vessels were visible during the arterial phase, with persistent enhancement during the venous phase. The second was an approximately 13 x 8 mm smooth endobronchial nodule in the left upper-lobe bronchus. An adjacent vessel was seen during the arterial phase, followed by persistent venous-phase enhancement. Both lesions had hypervascular features (Figure 1).

**Figure 2:**
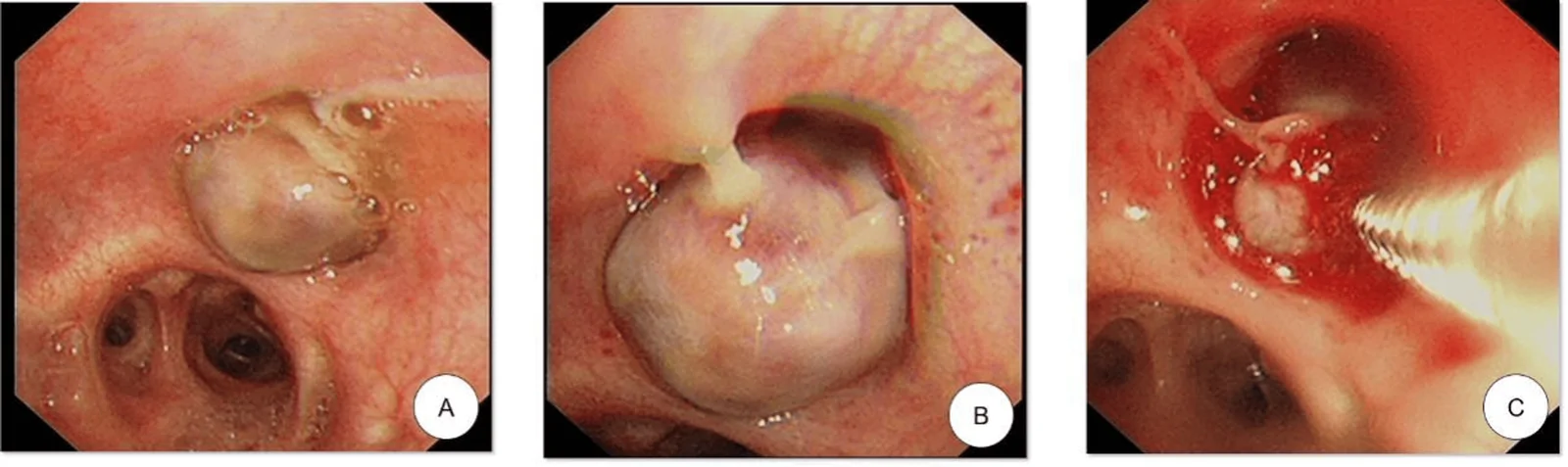
Electronic Bronchoscopy and Endobronchial Treatment. (A) Soft-tissue nodule in the left upper-lobe bronchus. (B) Smooth-surfaced nodule completely obstructing the bronchus. (C) Electrosnare resection of the mass under bronchoscopy.

Bronchoscopy was performed on hospital day 2. A smooth nodular mass was seen at the orifice of the left upper-lobe bronchus, completely obstructing the lumen (Figure 2A, B). A firm mass approximately 2 cm in diameter was removed with an electrosnare, with effective hemostasis. A small residual lesion was visible at the subsegmental opening of the proper segment of the left upper lobe and was treated by electrocoagulation (Figure 2C). Airway patency was restored, and the patient's vital signs remained stable.

Pathologic diagnosis and differential diagnosis

**Figure 3:**
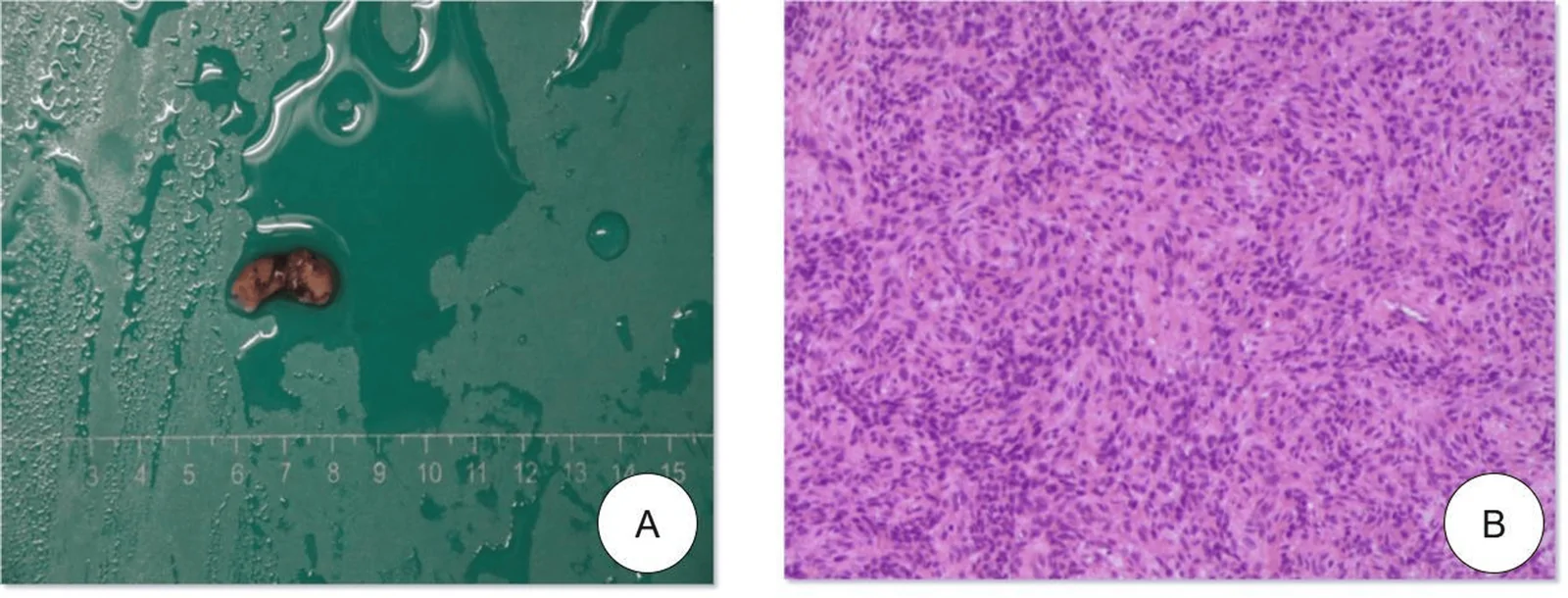
Bronchoscopic Resection Specimen. (A) Gross appearance of the mass. (B) Histologic appearance of the tumor showing oval cells with round-to-oval nuclei and a perivascular arrangement, consistent with a glomus tumor of uncertain malignant potential (hematoxylin and eosin stain, original magnification ×100).

The largest dimension of the bronchoscopically resected specimen was 2.3 cm. In some areas, the mitotic count was 4-6 per 10 high-power fields, and microscopy showed oval tumor cells. The pathologic diagnosis was glomus tumor of uncertain malignant potential (Figure 3). Although testing for NOTCH gene rearrangement was recommended for molecular characterization, it was not performed due to the patient's financial constraints.

On the basis of the symptoms and imaging findings, the preoperative differential diagnosis included tuberculoma, lung adenocarcinoma, and pulmonary sclerosing pneumocytoma. The patient had no low-grade fever, night sweats, or weight loss suggestive of tuberculosis, and no typical satellite lesions were seen. Both nodules had smooth margins without obvious spiculation, shallow lobulation, or mediastinal lymph node enlargement; nevertheless, malignancy could not be excluded given the endobronchial growth pattern. Hemoptysis, hypervascularity, and an adjacent-vessel sign can also occur in pulmonary sclerosing pneumocytoma; therefore, the final diagnosis depended on histopathologic examination.

**Figure 4:**
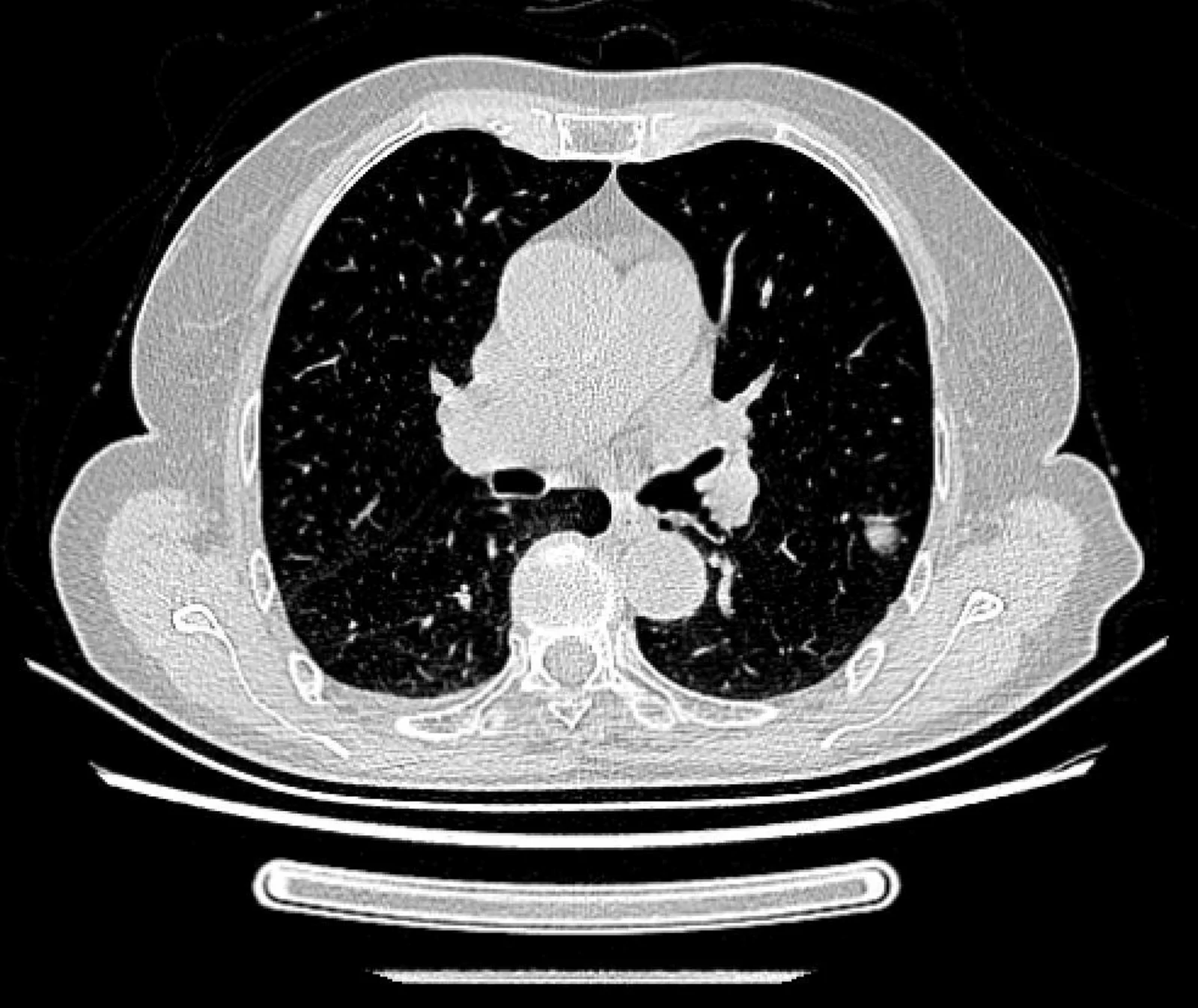
Noncontrast Chest Computed Tomography After Bronchoscopic Resection (Lung Window). The left upper-lobe bronchus is patent, with no definite residual endoluminal nodule.

Noncontrast chest computed tomography after bronchoscopic resection showed restoration of patency in the left upper-lobe bronchus, with no definite residual endoluminal nodule (Figure 4).

Therapeutic intervention

Because an independent parenchymal lesion remained, the endobronchial lesion was classified as a glomus tumor of uncertain malignant potential, and residual local disease was a concern, definitive surgery was selected after multidisciplinary review by radiology, pathology, and thoracic surgery. On hospital day 18, the patient underwent video-assisted thoracoscopic left upper-lobe sleeve resection with lymph-node dissection under general anesthesia. Extensive pleural adhesions were present, but there was no pleural effusion or pleural metastasis. After division of the left superior pulmonary vein and relevant pulmonary arterial branches, the left main bronchus was transected approximately 0.5 cm from the secondary carina and reconstructed with a continuous anastomosis. The underwater leak test was negative.

**Figure 5:**
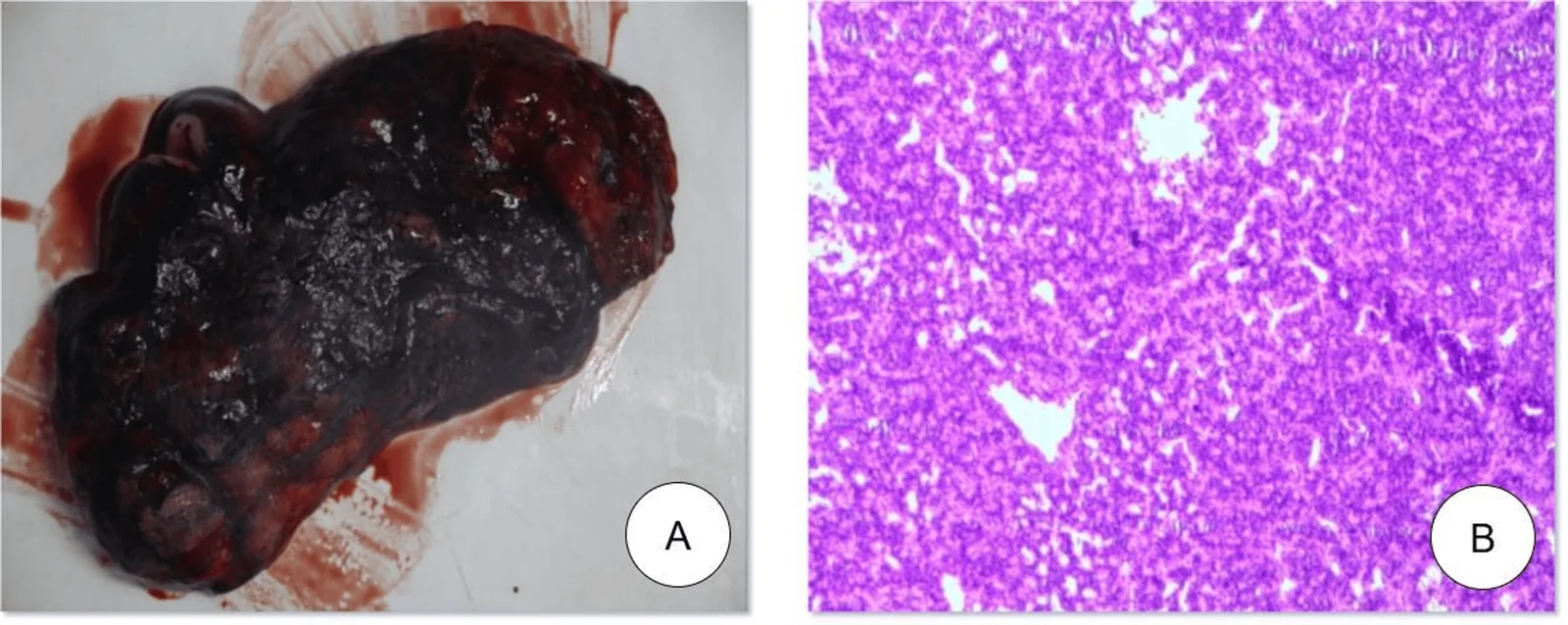
Left Upper-Lobe Sleeve Resection Specimen. (A) Gross appearance of the resected tissue. (B) Histologic appearance of the tumor showing uniform oval-to-round cells surrounding vascular channels, confirming the diagnosis of a glomus tumor of uncertain malignant potential (hematoxylin and eosin stain, original magnification ×100).

The resection specimen contained a 2.5 x 2.5 x 2.3 cm gray-white, well-circumscribed, friable mass adjacent to and protruding into the bronchus, approximately 0.3 cm from the pleura. Microscopically, the lesion was consistent with an endobronchial tumor and was diagnosed as a glomus tumor of uncertain malignant potential. The bronchial margins and visceral pleura were uninvolved, and no metastasis was identified in the dissected lymph nodes (Figure 5). 

Immunohistochemistry was positive for cytokeratin, desmin, smooth muscle actin, and vimentin, with partial positivity for CD56. TTF-1, p40, chromogranin A, synaptophysin, HMB45, Melan-A, S100, epithelial membrane antigen, ALK, and STAT6 were negative. CD31 and CD34 highlighted only vascular structures. The morphologic and immunophenotypic findings supported a diagnosis of glomus tumor (Table 2).

**Table 2:** Summary of the Patient's Immunohistochemical Staining Results and Related Clinical Significance. The patient's negative TTF-1 and p40 results, combined with imaging findings of smooth lesion margins and the absence of common malignant signs, strongly excluded the possibility of lung adenocarcinoma and lung squamous cell carcinoma. Ultimately, the positive SMA and Vimentin results, along with comprehensive clinical analysis, confirmed that the tumor exhibits the typical phenotypic characteristics of a glomus tumor. IHC: immunohistochemistry; SMA: smooth muscle actin; CK: cytokeratin; TTF-1: thyroid transcription factor-1; EMA: epithelial membrane antigen; CD: cluster of differentiation; ALK: anaplastic lymphoma kinase; NK: natural killer; SFT: solitary fibrous tumor.

Marker category	IHC marker	Patient's expression status	Common pathological significance
Mesenchymal/muscle tissue	SMA	Positive (+)	Smooth muscle actin. A marker for smooth muscle and myofibroblasts, and one of the typical positive expression indicators for glomus tumors.
	Vimentin	Positive (+)	A general marker for mesenchymal-derived tumors, and also a typical positive expression indicator for glomus tumors.
	Desmin	Positive (+)	A marker for muscle (including smooth and skeletal muscle) differentiation, suggesting myogenic characteristics of the tumor cells.
Epithelial and lung cancer differentiation	CK	Positive (+)	Broad-spectrum cytokeratin. Typically used to mark epithelial-derived tumors (e.g., various carcinomas), but can also be expressed in some mesenchymal tumors.
	TTF-1	Negative (-)	Thyroid transcription factor-1. A highly specific marker for primary lung adenocarcinoma and thyroid tumors. Its negative result in this case helps exclude lung adenocarcinoma.
	P40	Negative (-)	A marker for squamous cell carcinoma. Its negative result in this case helps exclude lung squamous cell carcinoma originating from the bronchial epithelium.
	EMA	Negative (-)	Epithelial membrane antigen. Commonly used to mark epithelial-derived tumors.
Vascular endothelial components	CD31	Blood vessels (+)	A specific marker for vascular endothelial cells, reflecting a rich vascular network within the tumor.
	CD34	Blood vessels (+)	A marker for vascular endothelium and hematopoietic stem cells, also indicating the presence of vascular components within the lesion.
Neuroendocrine differentiation	CD56	Partially positive (+)	A marker for neuroendocrine cells and natural killer (NK) cells.
	CgA	Negative (-)	Chromogranin A. A classic specific marker for neuroendocrine tumors.
	Syn	Negative (-)	Synaptophysin. A classic specific marker for neuroendocrine tumors.
Melanocytic/neurogenic differentiation	S100	Negative (-)	A general marker for neuroectodermal tumors (e.g., schwannomas) and melanomas.
	HMB45	Negative (-)	A specific marker for melanoma.
	MelanA	Negative (-)	A specific marker for melanoma.
Other specific tumor differentiation	ALK	Negative (-)	Anaplastic lymphoma kinase. Commonly used to screen for specific gene mutations in non-small cell lung cancer or large cell lymphoma.
	STAT6	Negative (-)	A highly specific marker for solitary fibrous tumor (SFT); a negative result helps exclude this rare tumor.

Outcome and follow-up

**Figure 6:**
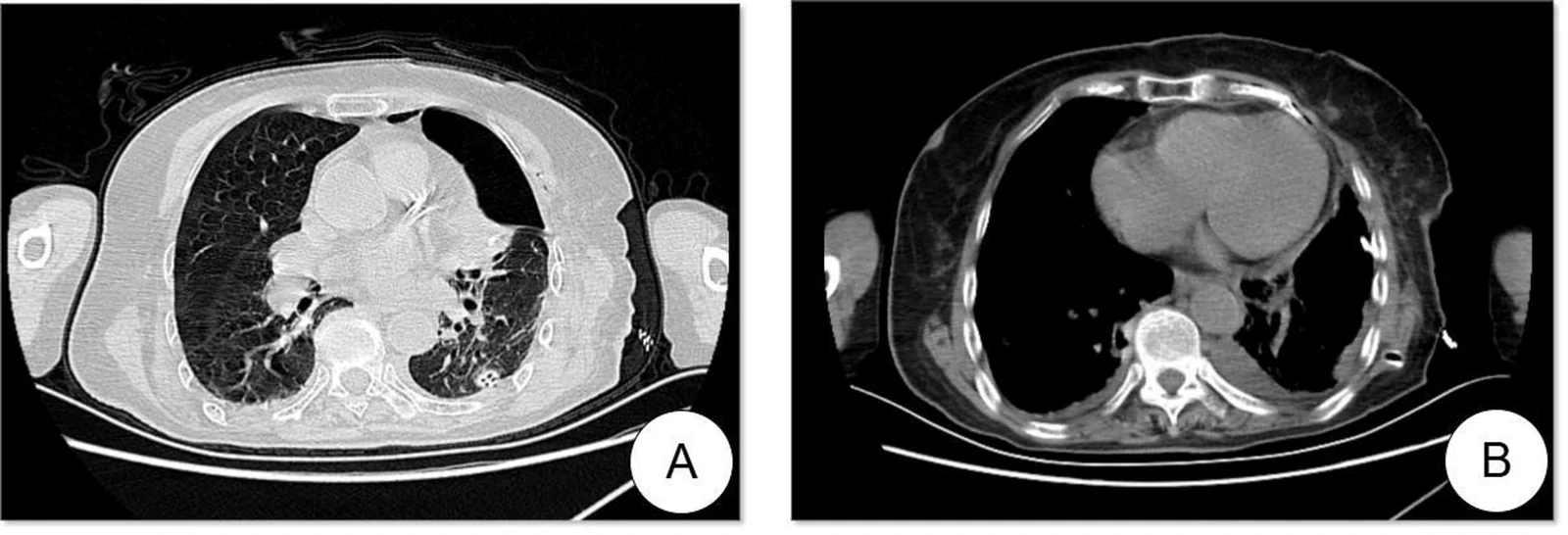
Noncontrast Chest Computed Tomography on Postoperative Day 2 After Left Upper-Lobe Sleeve Resection. (A) Lung window. (B) Mediastinal window. Left pleural air and fluid with a drainage catheter are present, consistent with postoperative change.

**Figure 7:**
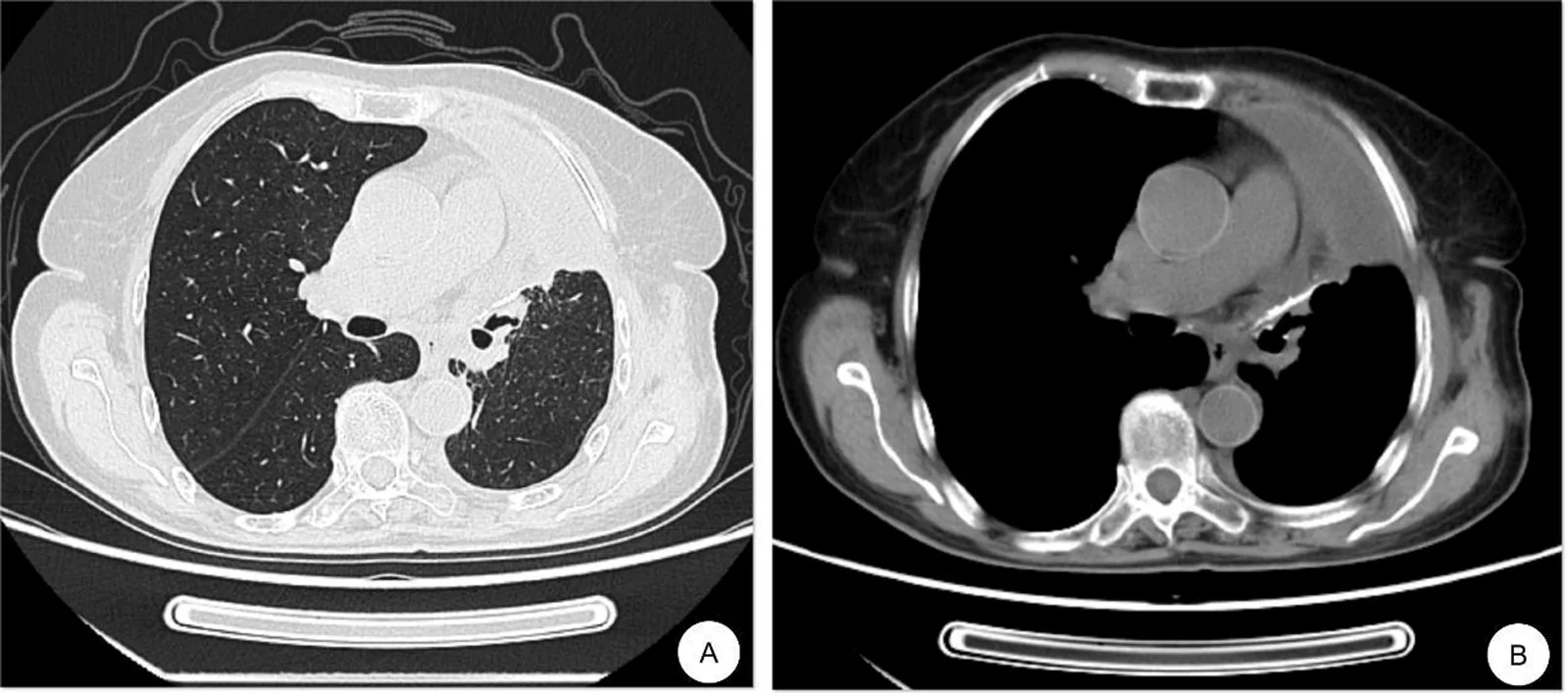
Noncontrast Chest Computed Tomography on Postoperative Day 23 After Left Upper-Lobe Sleeve Resection. (A) Lung window. (B) Mediastinal window. The left pleural air and fluid have decreased substantially compared with postoperative day 2.

Noncontrast chest computed tomography on postoperative day 2 after sleeve resection showed left pleural air and fluid with a drainage catheter in place, consistent with expected postoperative changes (Figure 6). A further examination on postoperative day 23 showed a substantial reduction in the extent of pleural air and fluid (Figure 7). The patient recovered uneventfully after both procedures. During the reported short-term follow-up, her condition remained stable and her quality of life was preserved; she continues to undergo regular surveillance. While negative TTF-1 and p40 render typical primary lung carcinomas less likely, it is the integration of the classic perivascular oval-cell morphology with the diffuse expression of smooth muscle actin, vimentin, and desmin that ultimately establishes the diagnosis of glomus tumor, despite the focal cytokeratin positivity. The patient has been followed up for 12 months with a plan for continuous clinical surveillance. Written informed consent was obtained, and the patient fully supports this ongoing monitoring strategy. 

## Discussion

Systematic evaluation of the existing global literature reveals an absolute scarcity of cases exhibiting simultaneous endobronchial and pulmonary parenchymal involvement. This coexisting pattern highlights an exceptionally rare manifestation among the limited number of reported pulmonary glomus tumors. In this case, both lesions were well circumscribed, hypervascular, and persistently enhancing, features that suggest a vascular lesion but are not diagnostic on their own. Previous reports similarly describe pulmonary glomus tumors as either polypoid endobronchial masses or parenchymal nodules, with final diagnosis usually established by pathology [1,2]. It is essential to first exclude primary lung carcinomas and typical benign vascular lesions when encountering a hypervascular endobronchial mass; pulmonary glomus tumor serves as a critical, albeit rare, secondary consideration. Clinicians evaluating hypervascular endobronchial masses with hemoptysis may include pulmonary glomus tumors in the differential diagnosis. This extreme rarity must not overshadow more prevalent diseases. The management of small intrapulmonary nodules relies on integrated clinical assessment. Serial low-dose chest CT provides necessary surveillance of growth dynamics. High-risk lesions are typically hypervascular. Any biopsy attempt requires rigorous prior evaluation of bleeding risks.

Imaging-based differential diagnosis should consider lesion location, margins, enhancement pattern, and associated findings. Tuberculoma should be assessed in the context of satellite lesions and clinical evidence of infection [4]. Lung adenocarcinoma may show vacuoles, spiculation, and pleural retraction [5]. Pulmonary sclerosing pneumocytoma can present with hemoptysis, marked enhancement, and an adjacent-vessel sign [6,7]. Although this case lacked typical infectious or overtly malignant features, the endobronchial component prevented exclusion of lung carcinoma before surgery. Recent evidence also indicates that some pulmonary squamous cell carcinomas can grow endobronchially; when imaging findings are discordant, prompt histologic confirmation is more reliable than morphologic inference alone [8,9].

An uncertain-malignant-potential subtype of tracheal glomus tumor has been reported [10]. Histologically, glomus tumors are usually composed of round or oval cells and commonly express smooth muscle actin and vimentin [9]. In this case, desmin was also positive, whereas CD31 and CD34 highlighted only vascular structures. Despite cytokeratin positivity and partial CD56 positivity, negative TTF-1, p40, chromogranin A, and synaptophysin results, together with the morphology, supported a glomus tumor and helped exclude epithelial and neuroendocrine neoplasms. This distinction is particularly relevant when endobronchially growing pulmonary squamous cell carcinoma is considered [8]. Particularly given the focal cytokeratin positivity observed, the definitive diagnosis of a glomus tumor cannot rest solely on negative markers but requires a holistic integration of the classic perivascular architectural patterns and the robust expression of smooth muscle actin (SMA) and vimentin. The bronchoscopic specimen exceeded 2 cm in greatest dimension, and focal mitotic activity was 4-6 per 10 high-power fields; the pathologist therefore classified the lesion as having uncertain malignant potential. Crucially, current risk stratification criteria are extrapolated almost entirely from soft-tissue glomus tumors. Given the extreme paucity of primary pulmonary cases, the prognostic validity of these criteria in the lung remains largely untested. For this reason, a designation of "uncertain malignant potential" inherently demands both exhaustive pathological sampling of the surgical specimen and long-term clinical vigilance. Pulmonary involvement by malignant glomus tumor has been described [11]. The criteria proposed by Folpe et al., including tumor size, location, nuclear atypia, and mitotic activity, may aid risk stratification; however, because pulmonary cases are sparse, classification and prognostic assessment should incorporate the complete resection specimen and long-term follow-up [12].

Treatment should balance symptom control, complete resection, preservation of pulmonary function, and potential malignant behavior. Bronchoscopic resection can rapidly relieve airway obstruction and yield diagnostic tissue. In this case, however, a small residual endobronchial lesion was suspected and an independent parenchymal lesion with the same pathology remained. The multidisciplinary team selected left upper-lobe sleeve resection. A left upper sleeve resection was meticulously planned by our multidisciplinary team for the following reasons: (1) the lesion demonstrated uncertain malignant potential (tumor size >2 cm and 4-6 mitoses/10 HPF); (2) residual disease persisted on bronchoscopic evaluation; and (3) there was direct anatomic continuity between the parenchymal mass and the bronchus. This specific surgical modality was chosen to achieve a complete R0 resection while sparing healthy pulmonary tissue that a conventional lobectomy would unavoidably remove. Importantly, performing this definitive surgery also aligned with the strong requests of the patient and her family, emphasizing the role of patient-centered care in our clinical practice. This achieved negative bronchial margins and pleura, with negative lymph nodes. Reports of pulmonary involvement by malignant glomus tumor underscore the importance of not overlooking high-risk pathologic features and the need for long-term surveillance, even though most glomus tumors behave benignly [11,12].

## Conclusions

Pulmonary glomus tumors can arise within the lung parenchyma or present radiologically as solid intrapulmonary nodules. Hypervascularity, persistent enhancement, and adjacent or tortuous vessels can provide diagnostic clues, but these features are nonspecific and cannot replace histopathology and immunohistochemistry. The clinical management of rare hypervascular pulmonary lesions, such as glomus tumors, necessitates prioritizing common etiologies, utilizing serial low-dose CT for nodule surveillance, and rigorously assessing hemorrhage risks prior to any biopsy.

When pathology indicates uncertain malignant potential, bronchoscopic resection carries a risk of residual disease, or a concomitant parenchymal lesion is present, management should be planned jointly by radiology, interventional pulmonology, pathology, and thoracic surgery. Definitive resection can provide complete pathologic assessment and reduce the risk of local residual disease. Long-term surveillance requires clearly documented follow-up time points.
